# Electrospun Nanofibers: Recent Applications in Drug Delivery and Cancer Therapy

**DOI:** 10.3390/nano9040656

**Published:** 2019-04-24

**Authors:** Rafael Contreras-Cáceres, Laura Cabeza, Gloria Perazzoli, Amelia Díaz, Juan Manuel López-Romero, Consolación Melguizo, Jose Prados

**Affiliations:** 1 Department of Organic Chemistry, Faculty of Science, University of Málaga, 29071 Málaga, Spain; rafcontr@ucm.es (R.C.-C.); amelia@uma.es (A.D.); jmromero@uma.es (J.M.L.-R.); 2 Department of Chemistry of Pharmaceutical Science, Faculty of Pharmacy, Complutense University of Madrid, 28040 Madrid, Spain; 3 Institute of Biopathology and Regenerative Medicine (IBIMER), Biomedical Research Center (CIBM), University of Granada, 18100 Granada, Spain; lautea@ugr.es (L.C.); gperazzoli@ugr.es (G.P.); jprados@ugr.es (J.P.); 4 Instituto de Investigación Biosanitaria ibs.GRANADA, 18012 Granada, Spain; 5 Department of Anatomy and Embryology, Faculty of Medicine, University of Granada, 18016 Granada, Spain

**Keywords:** electrospun nanofibers, cancer treatment, drug release, nanomedicine, biocompatible polymers, hyperthermia

## Abstract

Polymeric nanofibers (NFs) have been extensively reported as a biocompatible scaffold to be specifically applied in several researching fields, including biomedical applications. The principal researching lines cover the encapsulation of antitumor drugs for controlled drug delivery applications, scaffolds structures for tissue engineering and regenerative medicine, as well as magnetic or plasmonic hyperthermia to be applied in the reduction of cancer tumors. This makes NFs useful as therapeutic implantable patches or mats to be implemented in numerous biomedical researching fields. In this context, several biocompatible polymers with excellent biocompatibility and biodegradability including poly lactic-co-glycolic acid (PLGA), poly butylcyanoacrylate (PBCA), poly ethylenglycol (PEG), poly (ε-caprolactone) (PCL) or poly lactic acid (PLA) have been widely used for the synthesis of NFs using the electrospun technique. Indeed, other types of polymers with stimuli-responsive capabilities has have recently reported for the fabrication of polymeric NFs scaffolds with relevant biomedical applications. Importantly, colloidal nanoparticles used as nanocarriers and non-biodegradable structures have been also incorporated by electrospinning into polymeric NFs for drug delivery applications and cancer treatments. In this review, we focus on the incorporation of drugs into polymeric NFs for drug delivery and cancer treatment applications. However, the principal novelty compared with previously reported publications is that we also focus on recent investigations concerning new strategies that increase drug delivery and cancer treatments efficiencies, such as the incorporation of colloidal nanoparticles into polymeric NFs, the possibility to fabricate NFs with the capability to respond to external environments, and finally, the synthesis of hybrid polymeric NFs containing carbon nanotubes, magnetic and gold nanoparticles, with magnetic and plasmonic hyperthermia applicability.

## 1. Polymeric Nanofibers in Biomedicine: General Overview

The improvement of chemotherapeutic treatments in cancer patients is seriously limited by the difficulty of increasing the ability of drugs to specifically target tumor cells, thus reducing their toxicity in healthy cells. However, these antitumor molecules cannot increase their therapeutic response, which leads to poor prognosis and results in serious health problems, due to the prevalence of these several pathologies. Consequently, further research is needed to find new therapeutic approaches that improve the prognosis of patients affected by several types of cancers, thus decreasing the possible mortality rate. In this context, new strategies are appearing concerning the development of systems with the capability to encapsulate chemotherapeutic drugs and act as vehicles to be delivered in a specific area in a higher extension, thus avoiding the previously mentioned limitations and improving their efficiency, specifically in tumor cells [1,2]. According to the data from the World Health Organization, cancer caused 9.6 million deaths in 2018, and is one of the leading causes of death worldwide. The most common tumors are lung, breast and colorectal cancer [3]. For this reason, it is important to develop new nanoformulations that allow the improvement of cancer treatments and therefore the survival of these patients.

In general, drug delivery systems are nanostructures that can be loaded with small molecules or macromolecules, thus acting as vehicles of specific compounds to be used in a pharmaceutical administration process. Nowadays they represent one of the most promising challenges in the improvements of biomedical investigations [4]. Such materials are able to transport a chemotherapeutic molecule to a desired area, thus increasing the drug concentration, to be subsequently released in a controlled manner. Among a great number of nanoformulations, such as liposomes [5], micelles [6], Pickering emulsions [7], dendrimers [8] or nanoemulsions [9], polymeric nanoparticles (NPs) have been extensively reported as drug delivery systems to be applied in, for example, the chemotherapeutic treatment of solid tumors [9]. These NPs can be composed by synthetic polymers such as polylactic acid (PLA), poly lactic-co-glycolic acid (PLGA) or polyethylene glycol (PEG) [10,11], which in principle can be administered as colloidal systems and dispersed in a solution by means of intravenous administration. However, during the last years, apart from colloidal structures, polymeric nanofibers (NFs) have been reported as a scaffold with the ability to encapsulate antitumor drugs for biomedical investigations, including drug delivery and cancer treatments [12,13]. Among various techniques available for NFs fabrication, electrospinning is simple and produces NFs with high interconnected pores in the nanoscale range [14], having also a large surface area-to-volume ratio, high interfiber porosity, low hindrance for mass transfer, flexible handling, adjustable morphology, and high mechanical strength, which make NFs useful as therapeutic patches or mats for biomedical applications [15,16], indeed they are used in the fabrication of non-woven fibers with diameters ranging from a few nanometers to microns.

In the electrospun technique, when a strong electrostatic field is applied to a polymer solution held in a syringe, the pendent droplet of the polymer solution is deformed into a Taylor cone [17]. When the electric force overcomes the surface tension of the droplet, one or multiple charged jets are ejected from the tip of the droplet. As the jet moves towards a collecting metal screen, the solvent evaporates, and a nonwoven fabric mat is formed on the screen. This technique is able to fabricate fibers with diameter in the order of nanometers. During the last years, by using this technique, a great number of polymers have been used for the generation of biocompatible NFs scaffolds [18]. Biodegradable synthetic polymers such as PCL or PLGA, as well PEG and PLA can be used as substrates for the fabrication of NFs by using electrospun approaches [19]. Importantly, during the last few years, other types of polymers with stimuli-responsive behavior have been investigated as NFs scaffolds [20]. Two examples are poly(N-Isopropylacrylamide) (pNIPAM) and poly(4-vinylpyridine), which are the most investigated thermo- and pH-responsive polymers, respectively. By using this technique, the fiber diameter can be modulated by several polymer solution properties as viscosity, elasticity, polymer concentration and conductivity, the electric field strength, the distance between the injector and the metal collector, or other external parameters as temperature and humidity. Importantly, these NFs can incorporate and accumulate chemotherapeutic molecules by means of two main approaches [21]; (i) Blend electrospinning, which is based on mixing a drug with a polymeric solution prior to electrospinning process or (ii) Coaxial electrospinning, which is basically a simultaneous co-spinning of two polymeric liquids. The general system for this “core/shell” electrospinning is based on two needles, structured in a coaxial manner Indeed, drugs can be incorporated into the NFs through physical adsorption (involving electrostatic interactions) [22] or covalent bond [23], as well as the aforementioned co-axial electrospinning or mixing with the polymer solution. By applying some of the previously mentioned approaches, electrospinning also offers the possibility to fabricate hybrid composites NFs. These composites structures are produced by the incorporation of other systems with specific properties into the polymeric NFs as CNTs, magnetic nanoparticles or metal nanoparticles. Among several reported reviews concerning the fabrication of polymeric NFs by electrospinning for drug delivery purposes [24,25,26], the principal novelty of this review is that we include recent advances for the fabrication and application of polymeric NFs by electrospinning not only focused in polymeric NFs. We also include recent investigation in 3 major items: (i) the incorporation of particles as nanocarriers into the NFs (vesicles, micelles or silica particles), which are able to increase drug accumulations, (ii) the fabrication of polymeric NFs with the capability to respond to external environments, and (iii) the generation of hybrid systems structured as polymeric NFs containing CNTs, magnetic and gold nanoparticles for hyperthermia applicability.

## 2. Electrospun Nanofibers for Drug Delivery Application

### 2.1. Free drug-Loaded Nanofibers

**Figure 1:**
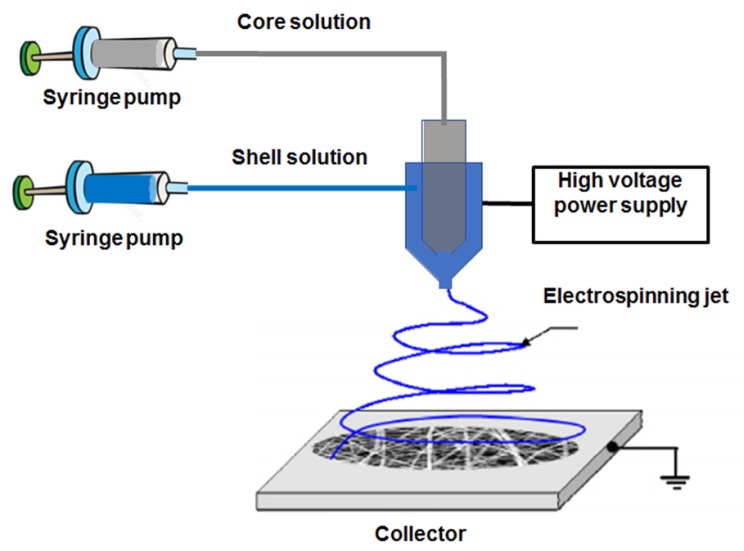
Schematic representation of the coaxial electrospinning setup. In this example the core solution is composed by the PTX dissolved in 2,2,2-trifluoroethanol and the shell solution is the poly(L-lactic acid-co-ε-caprolactone) polymer. Reprinted with permission from reference [27]. Copyright Wiley Online Library, 2009.

As was previously mentioned, coaxial electrospinning is based on co-spinning two liquids in a core/shell structure. Figure 1 shows a typical coaxial electrospinning setup used for the fabrication of core/shell NFs. As can be observed, it is composed by 2 syringe pumps. In this particular case, the injector is formed by a coaxial needle where the inner part contains a paclitaxel (PTX) solution and the outer part contains the polymer [27]. This technique has been applied for the incorporation of PTX into poly(L-lactic acid-co-ε-caprolactone), P(LLA-CL) (75:25)NFs, thus resulting in PTX loaded P(LLA-CL) NFs. Paclitaxel is a chemotherapeutic drug which is extensively used in breast, ovarian, lung, bladder and prostate cancer. Huang et al. [27] prepared a PTX solution by dissolving this chemotherapeutic drug in 2,2,2-trifluoroethanol. Then, co-axial electrospinning was used to directly introduce the PTX solution into a polymeric P(LLA-CL) shell. They prepared various core/shell NFs with tunable diameter, which was controlled by the polymer concentration and flow rate between the 2 solutions. Drug delivery investigations exhibited a short burst of PTX during 24 h followed by a very slow release for the following 60 days. Indeed, these PTX-IN-P(LLA-CL) NFs also inhibited the activity of HeLa cells. Paclitaxel was also introduced into PLGA nNFs mats fabricated by blend electrospinning for controlled drug delivery [28]. In this case, the drug release investigation was performed for the in vitro treatment of C6 glioma. The polymer fiber diameter was controlled by using different polymer concentrations, as well as different amounts of an organic salt named tetrabutylammonium tetraphenylborate (TATPB). They obtained NFs with dimension from several tens nanometers to 10 mm, and after the addition of organic salts, the NFs diameter were decreased up to 30 nm. For PTX-IN- PLGA NFs the encapsulation efficiency was higher that 90%. In vitro release profiles confirmed a sustained PTX release for more than 60 days. Their results also indicated that the density of C6 glioma cells was much lower after administration of different concentrations of PTX-IN-PLGA NFs, as compared to the control and blank PLGA NFs after 72 h.

Low-water soluble molecules as PTX are not the only option that has been introduced into NFs. A hydrophilic antibiotic drug, as MefoxinR, has also been incorporated into polymeric NFs by using PLGA and a mixture of PLGA/PEG-b-PLA/PLA (80:15:5) through blend electrospinning [29]. These authors demonstrated that the morphology and density of the prepared NFs depended on the drug concentration, which is basically produced by the different conductivity provided by the ionic salt during the electrospinning process. They obtained interesting antimicrobial effects on Staphylococcus aureus cultures when reaching a maximum dosage after 1 h. Indeed, when the amphiphilic block copolymer (PEG-b-PLA) with a ratio of 85:15 was used during electrospinning, the fabricated NFs were able to reduce the cumulative amount of the released drug at shorter times, and prolonged the drug release rate at longer times (up to a 1-week period). Blend electrospinning was also used for the encapsulation of three chemotherapeutic drugs into poly(L-lactide)(PLLA) NFs: PTX, doxorubicin (DOX) and DOX hydrochloride [30]. In this case, the influence of solubility and compatibility of drugs with the polymer was investigated, and it was associated with the drug delivery behavior. In these scaffolds, after drug incorporation, the degradation of PLLA fibers was monitored in the presence of the enzyme proteinase K, following a drug release with a nearly zero-order kinetics. Cisplatin is another compound that is used in chemotherapy for the treatment of liver cancer, although it has little effectiveness. Zhang et al. [16] used an electrospun system that consisted of five layers composed of PLA and the drug interleaved in layers. The drug was located in the even, second and fourth layers, while the PLA was located in the odd, first, third and fifth layers. In this study, fruitful results were obtained for in vivo investigations since the layered structure allows a continuous release of the drug. Consequently, a reduction in the toxicity during the treatment and a longer half-life of the mice was obtained. NFs can be also used for attenuating the side effects of the used drug. This is the case of the study of Singh et al. [31] These authors introduced Docetaxel (DOC) into polyvinyl alcohol (PVA) fiber using the electrospinning method. The aim of the study was to prevent inflammation, extravasation and other side effects of chemotherapy in the treatment of oral cancer. Because of that, they designed a mucoadhesive nano-carrier DOC-PVA and made in vitro studies with positive results, showing that anticancer drugs can be successfully used for local administration with polymeric NF.

Apart from chemotherapeutic drugs and antibiotics, Lovastain, a commonly used drug that reduces the cholesterol level and the risk of heart attack, has been incorporated into biocompatible NFs for drug delivery purposes. Zhu et al. [32] used blend electrospinning to introduce these biomedical properties of lovastatin into biodegradable and biocompatible PLLA NFs with drug delivery capability. Lovastatin was fully dissolved with PLLA by using hexafluoro-isopropanol as a solvent and at weight percentages of lovastatin in PLLA of 0%, 5%, and 10%. Interestingly, authors found that lovastatin values of 5 wt% or 10 wt% improved the NFs properties for alignment and surface smoothness, while also enhancing the NFs diameter. These Lovastain-IN-PLLA NFs reached high drug entrapment efficiency, ranging from 72% to 82%. The in vitro drug delivery investigations confirmed a release behavior in two stages. Initially, fast release was produced during the first day, and a slower release was measured that reached a plateau after 7 days. By using a cylinder collector during the electrospinning, they also fabricated PLLA films. These authors compared the drug delivery capabilities of PLLA NFs with PLLA films, and they found a higher release rate for fibers compared with films.

### 2.2. Nanocarriers-IN-Nanofibers

Nanocarriers have gained attention in drug delivery due to their ability to act as vehicles in the transport and delivery of different drugs. It is important to remark that nanocarriers are not only interesting in terms of vehiculization, they are also relevant because after introducing the drug into the nanocarrier, the amount of chemotherapeutic molecule to be delivered is considerably increased. It is accepted that a colloidal system is formed by a complex fluid where a certain substance (disperse phase) remains immersed in another substance (solvent). Accordingly, a colloidal system is formed by a disperse phase containing solid particles that are dispersed in a liquid. In this context, vesicles, micelles, microgels, or emulsions are colloidal structures where the disperse phase is a solid particle which is dispersed into a certain solvent. The possibility to introduce chemotherapeutic drugs into these colloidal particles has been extensively exploited in drug delivery applications and cancer treatments. Importantly, as these particles are in the range of nanometers, they can be incorporated into the human body by intravenous administration. However, it is important to mention that the simple introduction of drugs into some nanocarriers normally leads to inevitable burst drug release. To overcome this limitation, some improvements have been found, for example, by the incorporation of these nanocarriers into polymeric NFs, which improves drug delivery and the applicability of cancer treatments.

#### 2.2.1. Vesicles and Micelles as Nanocarriers

**Figure 2:**
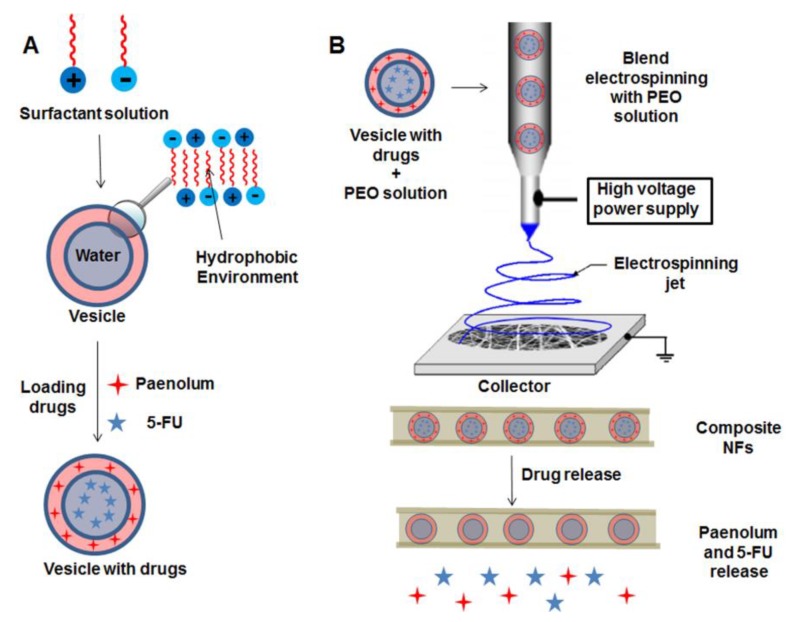
Schematic representation of the preparation of hydrophilic/hydrophobic electrospun composite fibers. (**A**) Accumulation of the drug mixture into the vesicle and (**B**) incorporation into the NF by electrospinning and drug release. Reprinted with permission from reference [33]. Copyright American Chemical Society, 2015.

Vesicles and micelles have been used as drug nanocarriers to be incorporated into polymeric NFs. An important advantage of this type of colloidal particles is the fact that they possess in their structure two different environments (hydrophobic or hydrophilic) that can be exploited in, for example, the incorporation of two different drugs, thus performing dual drug delivery. Li et al. [33] investigated a dual drug delivery approach by using two types of drugs, 5-FU and paenolum. 5-Fluorouracil is a hydrophilic chemotherapeutic drug principally used in colon cancer treatment, and paenolum is a hydrophobic molecule used to prevent blood platelet clotting, thus having anti-inflammatory properties. Initially, the vesicles were fabricated by mixing two surfactants, cetyltrimethylammonium bromide (CTAB) and sodium dodecylbenenesulfonate (SDBS), which were able to trap the aforementioned drugs. As is represented in Figure 2A, hydrophilic 5-FU were encapsulated within the aqueous inner part of the vesicle, and the hydrophobic paenolum was situated into the external bilayer of the vesicle. Then, after nanocarriers fabrication and drug encapsulation, they were mixed with a PEO solution, and blend electrospinning was performed to obtain a core/shell scaffold, with the drug-loaded vesicles as core introduced into PEO NFs, Figure 2B. The release investigations concluded that the hydrophilic drug was released in an increased manner when the molar ratio of CTAB/SDBS was higher. In contrast, the hydrophobic drug showed a decrease in the release capability as the molar ratio of surfactants was increased. Figure 2A,B shows a schematic representation for the incorporation of dual drug-loaded vesicles into PEO NFs. The vesicle is initially introduced into the mixture of drugs, and then electrospinning is used to form the vesicle-IN-PEO NFs scaffolds. More recently, the same authors fabricated three different vesicles that were used as nanocapsules systems introduced into polymeric NFs for drug delivery purposes. These vesicles were composed of didodecyldimethylammonium bromide, cetyl trimethyl ammonium bromide (CTAB)/sodium dodecyl benzene sulfonate (SDBS) (7/3) and CTAB/SDBS (3/7). After that, these vesicles were introduced into nanocapsules fabricated by a mixture of sodium alginate and chitosan. PEO NFs were obtained by blend electrospinning, and they were composed by PEO, containing a mixture of chitosan/sodium alginate with the vesicles as a template. In this work 5-FU was chosen as a model chemotherapeutic molecule to be incorporated during vesicles fabrication. The drug release behavior was followed by UV-visible spectroscopy. As was expected, the different drug-delivery systems showed different release rates and pH-responsive behaviors.

Other types of linear polymer, as pluronic F127, were also used for release investigations, along with with tissue regeneration. Electrospinning was chosen for the fabrication of a scaffold containing high molecular weight PCL NFs containing pluronic (F127) vesicles, which were delivered by exploiting the slow dissolution of PCL into glacial acetic acid. The vesicles were fabricated by pluronic F127 self-assembled with low-molecular weight PCL in a tetrahydrofuran-water mixture [34]. The authors were able to tune the vesicle size from 1 to 10 μm in diameter. Time-dependent stability of the vesicles in glacial acetic acid was determined before the electrospinning process. The electrospun membrane was found to be composed of pluronic F127/PCL vesicles within a PCL mat with a fiber diameter between 50–300 nm. Authors proposed that the most probable condition for the vesicles generation is the non-solubility driven self-assembly and stabilization of PCL and F127 into bilayers in the tetrahydrofuran (THF)-water mixture. By using this method, the amphiphilic polymer is dissolved in a water miscible organic solvent and mixed at a high speed with water, leading to the rapid precipitation of polymers into nanoscale particles. Drug delivery of a model molecule as rhodamine-B (introduced into the polymer network) for this composite pluronic F127-IN-PCL showed an important reduction in the release rate of this molecule, when it was compared to the free vesicles. Indeed, the systems containing vesicles in the membrane presented an enhanced hydrophilicity compared to the control PCL membrane. Apart from drug delivery behavior, this increased surface hydrophilicity was exploited for increasing the cell viability of L929 cells on the membrane [34]. Another study with PCL was carried out by Yohe et al. [35] using the N-38 anticancer drug for the colorectal cancer cell line HT-29. They electrospun a mesh using 10% of a hydrophobic poly(glycerolmonostearate-co-ε-caprolactone) (PGC-C18) and 90% of PCL loaded with the N-38, showing promising results in the cytotoxicity of the cell line.

Micelles introduced into polymeric NFs have been also used for dual drug delivery. Hu et al. [36] fabricated colloidal structures formed by a block co-polymer composed by methoxypoly(ethylene glycol)-block-poly(L-lactide). Into these colloidal particles, a water soluble chemotherapeutic drug (5-FU) and a lipophilic drug as cefradine (an antibiotic active against Gram positive bacteria) were introduced. The external shell of the NFs was fabricated by a mixture of chitosan and PEO, and the micelles loading drugs were introduced into NFs by blend electrospinning. The NFs without micelles were uniform and smooth, with diameters in the range of 100–500 nm. However, after drug-loaded micelles incorporation within the NFs, the surface of the NFs was relatively rough, with diameters in the range of 200–800 nm, with some black spheres with diameters of approximately 150 nm in the NFs. By using this drug loaded nanocarrier-IN-NFs, they performed release investigations that revealed a low burst release tendency of 5-FU and Cefradin. Indeed, this system was able to reduce the activity of HepG-2 cells with good cell viability after 3 days of incubation. Zhang et al. [37] fabricated micelles composed by triblock copolymers as poly(L-glutamic acid)-b-poly(propyleneoxide)-b-poly(L-glutamic acid) which were able to deliver low water-soluble drugs, such as PTX, at clinically relevant doses. These micelles containing PTX were used as drug loaded nanocarrier which were grafted onto NFs scaffolds of (poly(L-lactide-co-ε-caprolactone) (PLCL):fibrinogen; 2:1 (w/w)) by blend electrospinning. Yang et al. [38] fabricated an implantable device structured as drug-loaded micelles-IN-NFs for controlled drug delivery. In this case authors used DOX introduced into micelles composed by PCL-PEG copolymer. The implantable devices were fabricated by coaxial electrospinning, with the core composed by the DOX-loaded PCL-PEG micelles introduced into a PVA solution dissolved in distillated water. The outer shell layer of the NFs was composed by genipin cross-linked gelatin. Importantly, these authors also functionalized the micelles with folic acid to specifically target tumor cells. The drug release investigations demonstrated that the implantable device reduced the drug dose, the frequency of administration and side effect of chemotherapeutic drugs while maintaining highly therapeutic efficacy against solid tumors.

#### 2.2.2. Silica Particles as Nanocarriers

**Figure 3:**
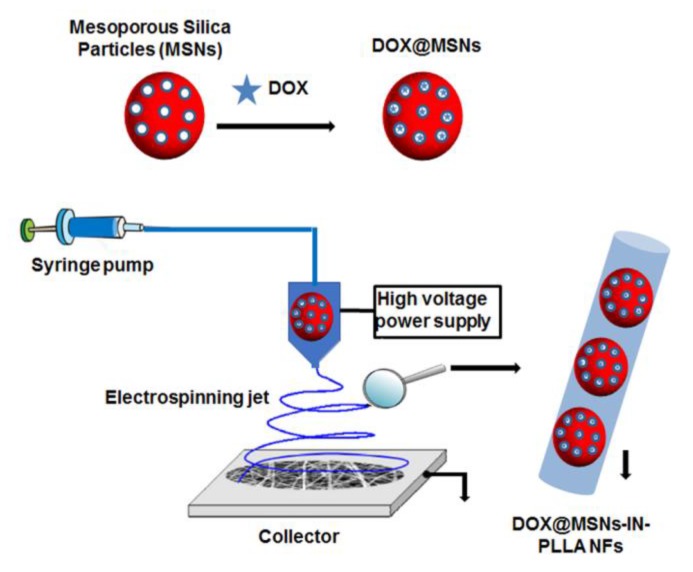
Schematic illustration for the process of fabrication of DOX@MSNs-IN-NFs electrospun composite NFs and the location of DOX in the fiber [40].

Dual drug delivery has been also performed by using two different types of colloidal nanoparticles as mesoporous silica nanoparticles (MSNs) and hydroxyapatite nanoparticles. These colloidal systems were used as nanocarriers for the incorporation of DOX HCl and the topoisomerase inhibitor hydroxycamptothecin [39]. Initially, the mixture of mesoporous silica particles and hydroxyapatite nanoparticles individually containing both chemotherapeutic drugs was a mixture within a polymer solution of PLGA to be introduced into NFs as a core/shell structure by electrospinning. This dual anticancer biocompatible system improved the mechanical capacity as well as the thermal stability of the NFs. In addition, when in vitro investigation was performed, the dual micelle-IN-NFs system provided a sustained and controlled drug release and an improved capacity for inhibiting HeLa cells growth. Using a similar approach, Qiu et al. [40] fabricated a drug-loaded implantable device for the treatment of a tissue defect after tumor resection. They used MSNs as nanocarriers for the incorporation of the anticancer drug DOX hydrochloride. These MSNs colloidal systems with incorporated chemotherapeutic drugs (DOX@MSNs) were introduced into PLLA NFs generated by electrospinning, thus obtaining a drug-loaded NFs scaffold DOX@MSNs-IN-NFs. Initially they confirmed the successful introduction of DOX-loaded MSNs into the PLLA NFs by UV-vis spectroscopy, and then several nanocomposite systems with different MSNs and DOX contents were fabricated. Figure 3 shows the schematic representation for the fabrication of DOX-loaded MSNs-IN-PLLA NFs. Optimal results concerning particles distribution that also improved thermal stability were found by using PLLA/1.0% DOX and 10% MSNs NFs. These authors investigated the in vitro antitumor efficacy against HeLa cells, and they found high DOX-loading capacities. Due to this fact, the drug was released in a sustained and prolonged manner, with a higher in vitro antitumor efficacy compared with free MSNs particles. Thus, these fabricated composite NFs mats are highly promising as a local implantable device for potential postsurgical cancer treatment. Yuan et al. [41] propose a drug delivery system which can release anti-tumor drugs in two phases. They designed a NFs scaffold for breast-conserving therapy after breast cancer by using DOX-loaded MSNs into an electrospun PLLA nanofibrous scaffold. In vivo results (mice) showed a significantly inhibition in the tumor growth, making its use promising in a coadjuvant therapy against this tumor type.

#### 2.2.3. Gelatin Nanoparticles as Nanocarriers

Song et al. [42] used gelatin nanospheres (GNs) as colloidal systems to improve the antibacterial effects of silk NFs membranes. They prepared two types of GNs by adding different amounts of glutaraldehyde into the suspension during the synthesis of GNs. The size and distribution of the GNs into the NFs, fabricated by electrospinning and using a mixture of PEO and silk fibroin, was monitored by fluorescent labeling. Positively charged drugs as vancomycin and colistin were used as a model for controlled drug delivery investigations. Vancomycin is an antibacterial compound that inhibits the synthesis of the bacterial cell wall, and colistin is an apolypeptide that is effective against most of the Gram-negative bacilli. These authors used both blend and coaxial electrospinning to fabricate GNs-IN-PEO/silk NFs, and they examined the antibacterial effect by introducing the mentioned antibacterial drugs. By using the NGs synthesized with the highest amount of glutaraldehyde, the fabricated NFs supplied a more sustained release of vancomycin compared with pure GNs. Indeed, apart from being totally cytocompatible, the NFs showed excellent and sustained antibacterial effects against Staphylococcus aureus. Lai et al. [43] fabricated collagen (Col) and hyaluronic acid (HA) NFs with the aim to release a series of growth factors directly embedded in the NFs or encapsulated in the gelatin nanoparticles (GNs) by using electrospinning technology. The fabricated GNs-IN-Col/HA NFs showed mechanical properties that mimicked human natural skin. The designed GNs-IN-Col/HA NFs were able to release growth factors in a slow controlled manner for up to 1 month. From the above, the electrospun Col-HA-GN composite nanofibrous skin substitute with a stage-wise release pattern of multiple angiogenic factors could be a promising bioengineered construct for chronic wound healing in skin tissue regeneration. They also used several release patterns for GNs-IN-Col/HA NFs with 4 different growth factors and demonstrated their potential capability to deliver multiple bioactive molecules.

Electrospun PLGA and PLGA/gelatin NFs embedded with MSNs were synthesized, obtaining MSNs distributed in the core of the fiber. PLGA and MSNs contributed to increase the hydrophobicity of electrospun NFs and the gelatin contributed to increase the mechanical properties of this scaffold. With a final size of approximately 267 nm, these nanoformulations are synthesized to provide a very suitable microenvironment for the adhesion, growth and migration of stem cells involved in nervous tissue regeneration [44,45]. Aytac et al. [46] synthesized electrospun gelatin NFs to vehiculate ciprofloxacin (CIP) and hydroxypropyl-beta-cyclodextrin (HPβCD)-inclusion complex (IC), which is normally used to improve the physico-chemical properties of some drugs as well as their bioavailability. This IC achieved an increase of the solubility and wettability of the nanoformulations, leading to their fast dissolution and therefore to a fast release of CIP transported in gelatin NFs, which can be an important property in certain situations where a rapid release of drugs is required. With this type of gelatin-based scaffolds, in addition to drug transport and use for tissue engineering, the migration of certain cells can also be induced or favored. This is the case in the study of Piran et al. [47] that synthesized electrospun three-layered scaffold with plasma enriched with growth factor to promote the migration and growth of fibroblast, which is a very important aspect in the regeneration of wounds.

#### 2.2.4. Stimuli-Responsive Nanoparticles as Nanocarriers

Some stimuli-responsive colloidal particles have been used as nanocarriers to be introduced into polymeric NFs for drug delivery applications. For example, Gong el al. [48] introduced redox-responsive nanoparticles into a polymeric scaffold to be incorporated into the body with the aim to deliver a growth factor (morphogenic protein BMP-2) used of bone regeneration. The strategy to fabricate redox-sensitive NFs with a core/shell structure consisted of a blend of PCL and redox responsive c-6A PEG-PCL nanogel with –S–S– bond on the outer shell. This redox-sensitive shell was able to respond to the change of the glutathione concentration and thus regulate the BMP-2 release for in vitro and in vivo investigations.

Light-responsive nanoparticles, as TiO_2_ and incorporated into a polyacrylonitrile (PAN)/multiwalled carbon nanotube composite NFs have been reported for the photocatalytic degradation of pharmaceutical molecules as Ibuprofen, Cetirizine, and Naproxen [49]. Visible light (0.1 W/cm^2^) irradiation was employed to investigate the drug degradation. The photocatalytic degradation of molecules using TiO_2_-IN-PAC/MWCNT NFs was higher compared with TiO_2_-IN-PAC NFs under visible light irradiation. A total degradation of drugs molecules was performed at 200, 50, and 90 min, respectively under visible light.

Another interesting strategy was developed by Elashnikov et al. [50]. They were able to release antimicrobial molecules from thermos-responsive microgels introduced into PLLA NFs. Crystal violet (CV) was incorporated within the polymer network of temperature-responsive PNIPAM microgels used as nanocarriers. Then, blend electrospinning was performed using PLLA as a biocompatible polymer, thus resulting in composite PLLA NFs with incorporated pNIPAM particles containing antimicrobial CV. They investigated the controlled drug delivery behavior of these core/shell NFs by UV-vis spectroscopy, which was produced after modification of the external temperature below and above the lower critical solution temperature (LCST). The antibacterial activity was investigated against gram-negative Escherichia coli (E. coli) and gram-positive Staphylococcus epidermidis (S. epidermidis). Authors demonstrated that the temperature-responsive release of antibacterial CV possessed remarkable antibacterial activity. This activity showed higher inhibition zones at temperatures above the LCST, with its size dependent on the polymers ratio and temperature.

### 2.3. Stimuli-Responsive Nanofibers

As was mentioned, synthetic polymers have been extensively used as scaffolds in the synthesis of NFs for drug delivery investigations. However, during the last years, important researching efforts have been focused on the use of stimuli-responsive systems [20]. These structures can undergo changes in response to external stimulus, as temperature, pH, ion strength, or solvent nature [51,52,53]. Most of the recent studies in the area of switchable drug release have been dedicated to the creation of systems for drug encapsulation based on two types of polymer [54]: pH-responsive and thermo-responsive polymers.

#### 2.3.1. pH-Responsive Nanofibers

The acidic environment found in tumor tissues can be employed as a way to specifically target the release of antitumor drugs at the tumor in response to a change in pH by the use of nanoformulations sensitive to pH changes [55]. Illangakoon et al. [56] used ES100, which is an anionic co-polymer constituted by metacrylic acid and methylmethacrylate, for the fabrication of pH-responsive nanofiber for the delivery investigation of 5-FU. Co-axial electrospinning was carried out with a core composed of poly(vinylpirrolidone), ethyl cellulose (EC) and the 5-FU drug, and the shell was formed from pH-responsive ES100. The drug release investigation demonstrated a controlled drug release developed at pH 1, reaching a maximum of 80% drug release after 2 h, produced by the diffusion of 5-FU through the pores of the ES100 polymer. At this pH, the polymeric fibers were fragmented, supplying an increased 5-FU delivery. Indeed, these authors fabricated NFs with a core/shell structure made of ES100 for the shell and Eudragit L100 (EL100) for the core to allow the controlled release at certain pH conditions, and controlled this release using the cover thickness [57]. Tran et al. [58] introduced ibuprofen into pH- and thermo-responsive polymers for controlled drug delivery investigations. Ibuprofen was initially mixed with a polymeric solution of PCL as the control experiment and also into poly(Nisopropylacrylamide-co-methacrylic acid) (pNIPAM-co-MAA) to fabricate stimuli-responsive NFs by blend electrospinning. As comparative results, when PCL NFs were investigated as a drug delivery system for ibuprofen, the fabricated NFs did not show significant drug release behavior at temperatures between 22–40 °C or pH from 1.7–7.4. However, NFs generated from pNIPAM-co-MAA were able to diffuse ibuprofen in a linear and controllable manner when the temperature was above the lower critical solution temperature (LCST) of pNIPAM-co-MAA (33 °C), as well as at pH lower that the pKa of carboxylic acids (pH 2). However, when the drug delivery experiments were performed at room temperature, the release rate was radically increased by closely ten times, compared to the release behavior at higher temperature and lower pH. NFs of cationic chitosan and poly(acrylic acid) (PAA) were synthetized with different levels of Cs deacetylation, showing that the mechanical properties of these NFs are determined by both the pH and the level of deacetylation, which could be useful in biomedical applications such as the transport and release of drugs [59]. Gelatin and poly(lactide-co-ε-caprolactone) (PLCL) were used to synthetize NFs that were loaded with ciprofloxacin and sodium bicarbonate with a response to low pH of gelatin/sodium bicarbonate fibers, whereas the hydrophobic PLCL had no sensitivity to pH [60]. These NFs not only showed good biocompatibility in fibroblasts (L929), they were also able to stimulate cell growth compared to untreated cells. Functionalized electrospun PCL scaffolds sensitive to pH changes, were loaded with DOX and tested at different pH levels (from 7.4 to 2.5) finding the highest drug release (90%–95%) at the lowest pH levels [61]. This was observed in the human embryonic kidney cells (HEK) treated with these scaffolds with a cell viability at pH 6 lower than those obtained at pH 7.2.

**Figure 4:**
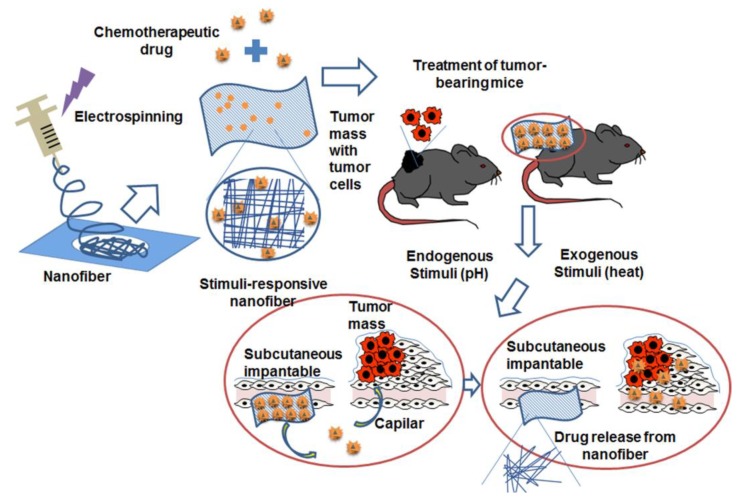
Stimuli-responsive NFs. Once NFs are synthesized by the electrospinning process and loaded with the antitumor drug, treatment may be applied in an experimental mouse model that carries a specific type of tumor. Once the treatment has been inoculated, an internal stimulus, such as the low pH present in the tumor tissues, or an external stimulus such as a temperature rise, stimulate the release of the drug at the specific site of the tumor, thus applying the treatment on tumor cells. Reprinted with permission from reference [62]. Copyright American Chemical Society, 2016.

Thixotropic silk NFs hydrogels were loaded with DOX and it was designed to release the drug in the tumor site due to their thixotropy capability [62]. This allows the hydrogel inoculation that then solidifies at the specific site and releases the drug in response to certain pH conditions. In the in vitro studies carried out on the human breast cancer cell line MDA-MB-231, it could be observed that the DOX-loaded hydrogels were more suitable for a long treatment, since even after 10 days they continued to inhibit cell growth, in contrast with free DOX. In the in vivo studies in breast tumors bearing BALB/c nude mice, it was observed that after the inoculation of the liquid hydrogel it solidified around the tumor, finding remains of the hydrogel even 5 weeks after the inoculation. In a similar way, in the in vivo studies after the inoculation, the decrease in tumor volume was similar with the treatment of free DOX and DOX transported by the hydrogels in the first weeks. However, at the fifth week, significant differences were observed in the volume and weight of the tumor treated with DOX-loaded hydrogels compared to free DOX, reaching reductions of approximately 1.5 times for both parameters, see Figure 4.

#### 2.3.2. Thermo-Responsive Nanofibers

In general, there are various protocols for synthesizing nanoformulations sensitive to temperature that are suitable for their possible use in biomedicine, because they can be administered by injection and may be degraded. These properties are useful in the transport of drugs for cancer targeting and controlled release such as degradable NFs fabricated by an electrospinning technique [63]. Slemming-Adamsen et al. [64] presented a novel approach to introduce DOX into thermoresponsive pNIPAM-NHS/gelatin NFs by cross-linking with 1-ethyl-3-(3-dimethyl-aminopropyl)-1-carbodiimide hydrochloride (EDC) and N-hydroxysuccinimide (NHS). This strategy consisted of a mixture with a solution of pNIPAM-NHS/gelatin acting as a shell with another mixture of EDC and NHS in the presence of DOX. By using this approach, EDC initiates the conjugation by bonding with a carboxyl group of the polymer. Then, the EDC-polymer conjugate is able to react with a primary amine, or, NHS, replacing EDC with the amine ester linkage. Finally, NHS can be replaced by a primary amine, linking the carboxyl-polymer with the amine-polymer. This mixture was electrospun to obtain cross-linked pNIPAM/gelatin NFs containing an anticancer drug that can be released in a controlled manner. The DOX-IN-pNIPAM NFs showed thermo-responsive swelling/deswelling properties. Indeed, the fabricated cross-linked NFs were able to release DOX when the temperature was raised above the LCST and were able to reduce the viability of human cervical cancer cells [64].

Zhang et al. [65] fabricated a core/shell structure formed by polylactic acid PLA as a core using electrospinning, and then a thermoresponsive pNIPAM shell was incorporated by UV photo-polymerization. Initially, biodegradable PLA NFs were fabricated by electrospinning in the presence of Combretastatin A4 (CA4), a tubulin polymerization inhibitor which was used as the model drug was produced. These fabricated PLA NFs were introduced into a pNIPAM solution in presence of the crosslinker (*N,N′-*methylenbisacrylamide). After exposed to UV radiation, the drug-loaded PLA core was coated and cross-linked with a pNIPAM shell. The composite NFs exhibited different wettability confirmed by water contact angle measurements at temperatures below or above the lower critical solution temperature (LCST) of pNIPAM. Most importantly, in vitro drug release investigations demonstrated a difference drug release when the temperature was at 25 or 40 °C. For example, the pNIPAM shell could limit the release rate of CA4 below the LCST, however, above the LCST, the rate of drug release increased significantly. Cicotte et al. [66] used thermos-responsive pNIPAM films fabricated by electrospinning to exploit a rapid reversible adhesion of mammalian cells, thus performing cell attachment and detachment using pNIPAM scaffolds. These authors modified various parameters during the electrospinning process such as the needle gauge, collection time, and molecular weight of the polymer. Two types of cells were investigated for reversible attachment of pNIPAM mats that provided potential results by seeding mammalian cells from standard cell lines (MC3T3-E1) as well as cancerous tumor (EMT6) cells. Once attached, the temperature of the cells and mats was changed to ~25 °C, resulting in the extremely rapid swelling of the pNIPAM NFs. The authors found that pNIPAM mats fabricated using small and dense fibers fabricated from high molecular weight pNIPAM polymers are extremely appropriate as a rapid release method for cell sheet harvesting. Recently, new nanoformulations that allow the release of drugs in a dual way in response to both temperature and pH stimuli have been designed. This is the case of poly(N-isopropylacrylamide-co-acrylic acid) NFs in a passive thermoplastic polyurethane (TPU) which are sensitive to pH and temperature. Consequently, by varying these two parameters, the movement in terms of direction and size can be modulated, which could be interesting in several biomedical applications, such as drug release [67]. In another study, a thermo-sensitive polymer, PNIPAAm and a pH-sensitive polymer, Eudragit^®^ L100-55 (EL100-55), were synthetized and made NFs by electrospinning [68]. These NFs showed sensibility to pH and temperature and a release of ketaprofen that are dependent on these parameters without toxicity against fibroblast, even at high concentrations. Another example is a fiber mixture of poly(N-vinylcaprolactam) and ethyl cellulose (EC) in the case of temperature-dependent release and EL100 fibers for pH-dependent release, synthesized by twin-jet electrospinning [69]. This mixture of fibers showed a sustained release of the non-steroidal anti-inflammatory drug ketoprofen in response to pH and changes in temperature, also showing a very good biocompatibility in fibroblasts. The study of biocompatibility of these NFs is noteworthy because it is an essential property that nanomaterials which are intended for therapeutic use must comply with. For this purpose, fibroblasts (L929) were seeded on cover slips that were previously sterilized and where fibers were directly slectrospun. After 1, 3 and 5 days of exposure, cytotoxicity is determined by the MTT assay. NFs are inclined to show good biocompatibility, but some types may be more appropriate than others, such as thermosensitive fibers made of poly(di(ethylene glycol) methyl ether methacrylate) (PDEGMA) synthesized and electrospun into fibers using EC, which showed great biocompatibility even after 5 days of exposure and better in vitro biocompatibility than other nanoformulations such as EC/NIPAM [70,71]. Another type of stimuli-responsive NFs that could be used for the transport of antitumor drugs is electrospun self-immolative polymer (SIP)/polyacrylonitrile (PAN) fibers [72], which depolymerize surprisingly rapidly in response to an external stimulus, producing an instantaneous release of the transported molecules at the right time.

### 2.4. Hybrid Nanofibers

#### 2.4.1. CNTs/Nanofibers

**Figure 5:**
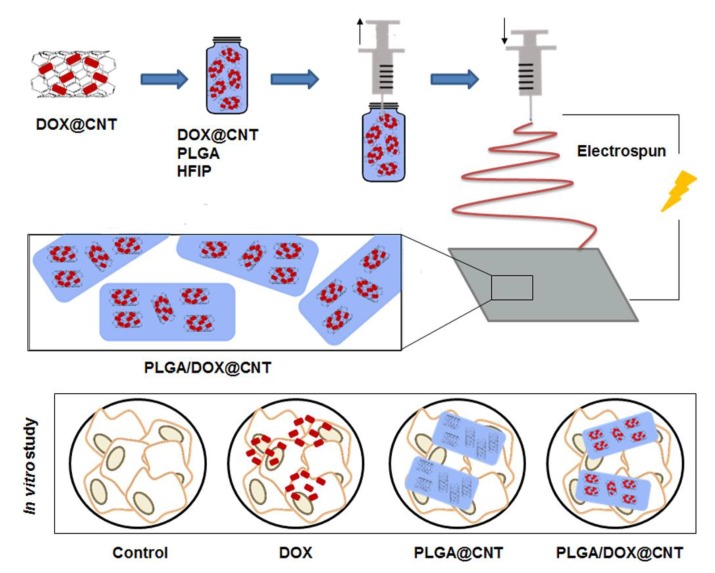
Schematic illustration for the fabrication process of PLGA/DOX-IN-CNTs electrospun composite NFs. The morphology and diameter distributions of PLGA and PLGA/DOX@CNTs composite NFs [74].

Carbon nanotubes have been used as platforms to improve mechanical, structural and drug delivery properties in NFs synthesized by electropun. In a recent investigation carried out by Qi et al. [73], DOX was chemically incorporated onto the surface of MWCNTs. After being optimized, the drug encapsulated up to 83.7% and the dispersion of MWCNTS@DOX particles was mixed with a PLGA polymer solution at 3 different amounts of DOX, relative to PLGA, to fabricate a composite NF by electrospinning. Importantly, the incorporation of MWCNTs into PLGA NFs did not alter the structure of the PLGA NFs, instead it improved their mechanical properties. In vitro viability assay demonstrated that the developed DOX-loaded MWCNTs-IN-PLGA composite NFs were totally cytocompatible with L929 cells. The drug delivery investigation confirmed that this composite system was able to reduce burst DOX release, and it also allowed a continued DOX release over 42 days. This composite structure structured as CNTs-IN-NFs was also used for Yu el al. for the incorporation of DOX HCl thus creating a hybrid NFs mat by blend electrospinning. In this case CNTs were also used as carriers of DOX and they were introduced within PLGA NFs by electrospinning, thus creating a composite nanofibrous mat. In this investigation, authors modified the amount of CNTs in the final mixture polymer-CNTs during electrospinning. The in vitro antitumor efficacy against HeLa cells was investigated, resulting in a DOX release by a sustained and prolonged manner, which effectively inhibited growth of HeLa cells. Figure 5 shows a schematic representation for the fabrication of DOX-loaded CNTs-IN-PLGA NFs by blend electrospinning.

Zhang et al. [75] fabricated a new class of highly porous NFs by using PLA as the principal polymer scaffold and PEO as a porogen. Different concentrations of CNTs were incorporated onto the fibers to allow self-sealing behavior, which was carried out by photothermal conversion after light irradiation. Basically, they developed a strategy to fabricate porous PLA/CNT fibers with controlled pore sizes. Indeed, CNTs were used in the current study to perform the pore self-closure through their photothermal conversion ability. Zhang et al. investigated several ratio polymer/CNTs during the electrospinning process and they found that the fibers containing 0.4 mg/mL of CNTs showed the optimum encapsulation efficiency of model biomacromolecules such as dextran, bovine serum albumin, and nucleic acid. The pores of the surface were reversibly reopened by PLA degradation, reaching a stable release of biomacromolecules after encapsulation. They showed morphological changes of PLA/CNT fibers with 0, 0.2, 0.4, and 0.8 mg/mL CNT concentrations. At a CNT concentration of 0.2 mg/mL, only a small amount of CNTs could be seen on the fibers surface. As the concentration was increased to 0.4 mg/mL, much more CNTs were observed, which seemed to be dispersed homogeneously around the nanopores on the fibers. Nevertheless, with further increase of the CNT concentration to 0.8 mg/mL, significant agglomeration was observed. After trapping the drug molecules, a controlled and sustained release from the fibers over extended periods of time was investigated. An extended release of the molecules for over the period of 15 days was achieved, with a relatively low initial burst release within the first 24 h. Indeed, in these structures the amount of CNT introduced into the nanofiber was relevant to control parameters as morphology, structure, thermal/mechanical capability, degradation, and cell viability.

**Figure 6:**
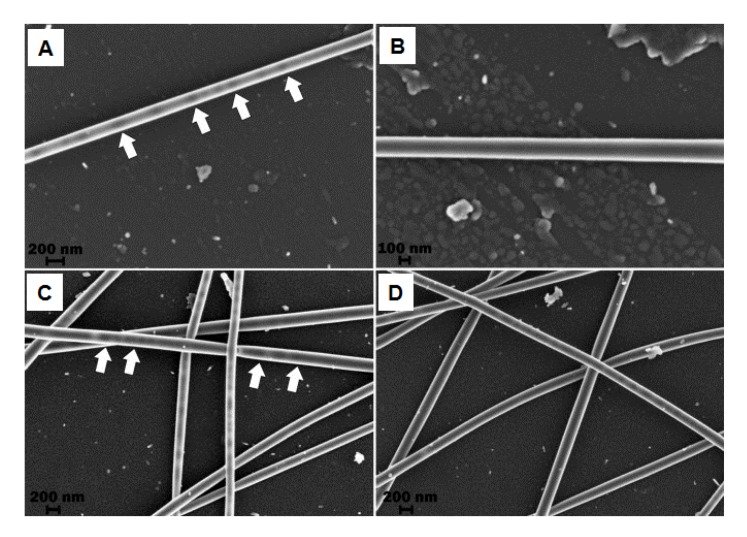
SEM micrographs of PVA/(graphene foam and expanded graphite) NFs at 2 kV operating voltage for (**A**) solution with 0.02 g GF concentration, (**B**) solution with 0.08 g GF concentration, (**C**) solution with 0.02 g EG concentration and (**D**) solution with 0.08 g EG. Reprinted with permission from reference [77]. Copyright Elsevier, 2015.

It is well-known that CNTs have the capacity to acts as thermal generators by absorbing near-infrared radiation (NIR). Zhang et al. [76] combined MWCNTs and DOX in a methanol/chloroform solution with PLLA dispersed in CHCl_3_ to fabricate NFs by the electrospinning technique. Without NIR irradiation, DOX release was extremely restricted. However, when NIR irradiation (2 W/cm^2^) was applied on DOX-loaded MWCNTs-IN-PLLA NFs burst release of the loaded DOX was observed at the time of 2 h with an amount of ~20% during the 30 min irradiation period. Indeed, these authors demonstrated that after NIR radiation, the temperature of the tumor area in contact with the NFs was significantly increased. Apart from that, these multifunctional NFs showed increased cytotoxicity both in vitro and in vivo for Hela cancer cells through the combination of photothermal induced hyperthermia and drug delivery. Barzegar et al. [77] introduced graphene into PVA NFs by electrospinning, thus creating grapheme/PVA hybrid NFs. Figure 6 shows scanning electron microscope (SEM) images where uniform hollow PVA NFs containing graphene dispersion within the NFs can be appreciated. The synthesized polymer reinforced NFs have potential biomedical materials for drug delivery.

In recent years, there has been an increase in the number of investigations related to the use of electrospun NFs combined with conductive nanomaterials, as biosensors, due to their promising applications [78]. Electrospun NFs in combination with CNTs have also been investigated as biosensors for the detection of early stages of pancreatic cancer by the use of the biomarker CA19-9. Electrospun NFs made of poly(allylamine hydrochloride) and polyamide 6 were covered with MWCNTs, and CA19-9 detection were determined by electrochemical impedance spectroscopy [79]. This biosensor showed a good sensitivity for the antigen without the rest of the blood components could suppose interference in the detection. This high sensitivity was achieved through antibody-antigen irreversible adsorption. In addition, this system was able to distinguish between patients with higher levels of the biomarker in blood and those who had it in lower concentration.

However, concerning toxicology studies, it has been reported that CNTs produce some adverse effects, due principally to the incorporation of catalytic metal impurities during their fabrication [80]. For this reason, a general use of CNTs in the biomedical field has generated some doubts about their security and possible toxicity. For example, long-term accumulations of CNTs were produced in lungs and liver of mice after exposure during 90 days [81]. Some reported investigations detailed the negative effects of these CNTs in biological systems. An alternative to reduce toxicity is the use of surface functionalized CNTs. Specifically, a recent investigation showed that pure CNTs had negative effect on the systemic immunity, producing more inflammation and immunosuppression as compared to some surface modified CNTs, such as PEG-modified CNTs [82].

#### 2.4.2. Magnetic Nanoparticles/Nanofibers

Magnetic nanoparticles are another type of colloidal particles widely used in biomedical applications. The systems, in the range of nanometers, are able to respond to external magnetic fields. This property has been extensively exploited in the biomedical field for localized drug delivery, because they can be transported to a specific area by the application of an external magnetic field. Apart from conventional drug delivery systems used in nanomedicine for the reduction of solid tumors, magnetic hyperthermia treatments [83,84,85], in which magnetic or magnetic-derived NPs are administered to tumors, and then a local heat is supplied under alternating magnetic field (AMF) application, have attracted an ever increasing interest due to their improved precision for cancer therapy [86,87,88]. The basis of using hyperthermia as a treatment modality for cancer is the high sensitivity of cancer cells to temperatures in the range from 41 to 45 °C, in contrast to normal cells. However, the use of free magnetic nanoparticles has certain limitations such as poor tumor targetability, high variability in the amount of magnetic nanoparticles administered to the tumor, as well as the transfer of magnetic nanoparticles into the healthy tissues close to the tumor. To overcome this disadvantage, magnetic NPs have been also used in electrospinning to fabricate hybrid NFs for drug delivery and cancer treatment purposes.

It is important to mention that a significant advantage of magnetic nanoparticles is that they present a reduced toxicity, while also being accepted by the human body. Indeed, once they are placed into the cells they degraded reasonably quickly [89]. The degradation of magnetic nanoparticles into iron and oxygen is performed inside the lysosomes of macrophages, and it is influenced by several parameters as the presence of hydrolytic enzymes, a low pH, as well as proteins related with the iron metabolism. In particular, iron oxides nanoparticles are able to degrade in vivo by iron mobilization and some other published routes [90]. Most importantly, magnetite is an iron oxides derivative approved by FDA for in vivo investigation [91].

**Figure 7:**
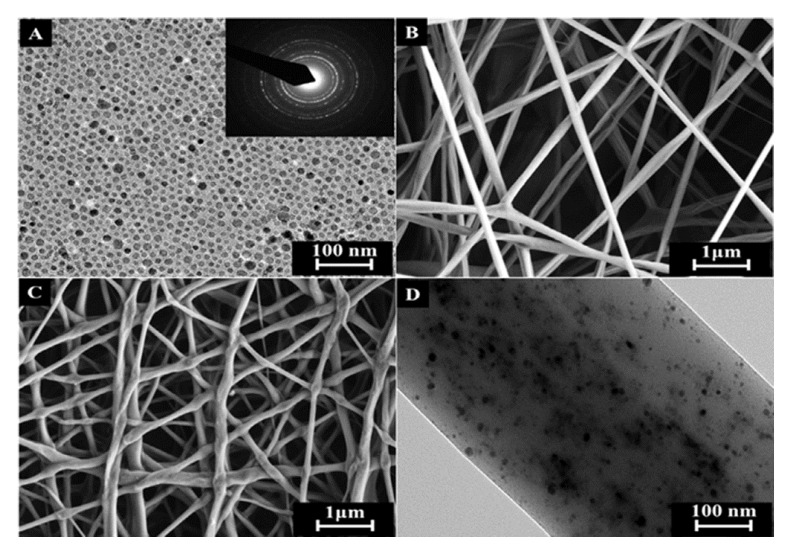
(**A**) TEM image of the iron oxide NPs (the inset shows the corresponding selected-area electron diffraction pattern), (**B**,**C**) FESEM images of electrospun PLGA (poly lactic-co-glycolic acid) NFs and magnetic NF matrix, respectively, (**D**) TEM image of MNF. Reprinted with permission from reference [94]. Copyright Elsevier, 2016.

Feng et al. [92] prepared a mixture of Fe_3_O_4_ NP and graphene oxide sheets containing functional groups to be introduced into PAN NFs for guiding cellular application. They used blend electrospinning to fabricate a hybrid GO/Fe_3_O_4_-IN-PAN as short-fibers (SFs). These synthesized NFs films were cut into small pieces, and after being dispersed in tert-butanol solution, they demonstrated a strong magnetic capability. As guiding cellular behavior, breast cancer cells were cultured on the surface of these magnetic SFs, and due to the external GO on the surface of SFs they promoted adhesion of cell membrane proteins and good biocompatibility. Indeed, guided cellular behavior by magnetic actuation with the help of magnetic SFs was performed. Huang et al. [93] introduced 50 nm iron oxide nanoparticles into polystyrene (PS) NFs scaffolds. Fe_3_O_4_ NPs were initially dispersed and ultrasonicated in THF, this dispersion was a mixture with a PS solution to proceed with blend electrospinning. Upon applying an AMF on these hybrid Fe_3_O_4_-IN-PS NFs, an important heating process occurred due to the high loading capacity of the fibers. Using this hybrid structure, the fabricated magnetic fibers can be heated several times without loss of heating capacity or releasing magnetic nanoparticles. Indeed, these authors functionalized the surface of the hybrid NFs structure with collagen in order to increase cell attachment. In vivo investigations were performed by using Human SKOV-3 ovarian cancer cells, which were incorporated onto the fibers. After the application of an AMF during 10 min to the mats, the cancer cells deposited on the Fe_3_O_4_-in-fibers were eliminated. This methodology possesses two important advantages to be implemented for in vivo investigations, as the fibers can be loaded with magnetic nanoparticles in a controlled manner and the composite scaffolds can be localized in the body by magnetic resonance imaging (MRI). Sasikala et al. [94] fabricated an implantable hybrid magnetic NFs device to be applied in both magnetic hyperthermia, upon an AMF, and cancer cell-specific drug release, to perform a synergistically cancer therapy. A borate-containing anticancer drug was investigated known as bortezomib (BTZ), a protease inhibitor frequently used in chemotherapeutic administration. This device was fabricated by blend electrospinning, by a mixture that initially was a Fe_3_O_4_ NPs dispersion and a PLGA solution. Then, a shell of polydopamine was grown through a simple immersion method to be used as a shell-mimicking mussel adhesive. The mussel-inspired magnetic NFs with numerous catechol moieties were able to bind and release borate-containing anti-cancer drugs. Figure 7 includes transmission electron microscopy (TEM) image of free Fe_3_O_4_ NPs as well as field emission scanning electron microscopy (FESEM) and TEM images of electrospun PLGA NFs. The FESEM images confirmed that hybrid NFs shows good fiber morphology and some aggregation of the Fe_3_O_4_ NPs inside of the NFs. They investigated the effect of repeated hyperthermia application in murine breast cancer (4T1) cell lines by using the BTZ-loaded Fe_3_O_4_-IN-PLGA NFs. After three hyperthermia cycles (15 min/24 h) were applied to the BTZ-loaded Fe_3_O_4_-IN-PLGA NFs, an improved antitumor efficacy was obtained.

In other reported cases, polyurethane NFs combined with superparamagnetic iron nanoparticles (γ-Fe_2_O_3_) have been synthesized for cancer treatment by hyperthermia, which were able to reach a temperature increase of up to 43 °C in 70s by the application of an AMF. This promising result was achieved by making the electrospinning process much more precise with the addition of a conical aluminum auxiliary electrode [95]. Radmansouri et al. [96] designed DOX hydrochloride-loaded electrospun chitosan/cobalt ferrite/titanium oxide NFs to treat melanoma cells (B16F10) by means of chemotherapy and hyperthermia at the same time. NFs were made of chitosan by the electrospinning process and were combined with cobalt ferrite nanoparticles and titanium oxide nanoparticles (used to modulate the temperature increase). Higher cell death was observed when hyperthermia and chemotherapy were combined, achieving a synergistic effect, which enhances the cytotoxic effect and allows a reduction of side effects.

#### 2.4.3. Gold Nanoparticles/Nanofibers

It is well-known that some non-spherical gold nanoparticles (Au NPs) display large near infrared (NIR) resonances that can be used to induce both hyperthermia and drug delivery when they are irradiated with the appropriate wavelength [97]. As non-invasive therapy, NIR radiation is crucial for biomedical uses because it penetrates tissue more deeply, but it is absorbed less than other types of radiation. NIR hyperthermia is a minimally-invasive oncological treatment strategy in which photon energy is selectively administered and converted into sufficient heat to induce cellular injury [98,99,100]. An important property concerning Au NPs is that by varying their size and shape, the surface plasmon absorption can be tuned from ultraviolet (UV) to infrared (IR) wavelengths. Recently, the potential uses of gold nanoparticles in NIR-hyperthermia have been reported using a variety of noble metal nanostructures, including gold nanoshells [99,101], gold nanorods [102,103,104], and recently, gold nanocages [105]. The potential toxic impact of AuNPs has been discussed. The particles size, surface chemistry and the presence of functional groups may play a relevant role in cell toxic effects [106]. Some studies have reported that cationic Au NPs are toxic while anionic AuNPs are non-toxic for cells [107]. The toxicity is caused by the electrostatic interaction of Au NPs with the negatively charged bilayer of the cellular membrane. Several investigations have also confirmed that some modified Au NPs (as PEG-modified Au NPs) are non-toxic at the dose that is effective for in vivo drug delivery [108]. Recently, the synthetic toxicity of AuNPs capped with polyethylenimine (PEI) and PEGylated anisamide has been tested for in vivo investigations, obtaining changes in blood cells by hemocytometer. These results demonstrated nonsignificant differences between hematological toxicity of these modified NPs and controls (saline serum) In addition, an extensive analysis of the tissue injury was carried out using gold nanoparticles prepared with PEG and DOX. In this case, Au3 treatment did not induce histopathologically observable differences in mice (including among others, heart, lung, stomach, intestine, liver, pancreas, kidney, spleen, skeletal muscle, brain, spinal cord) from those treated with saline serum, thus indicating no systemic toxicity [109].

**Figure 8:**
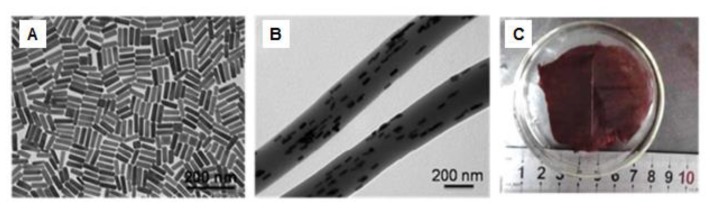
TEM images of (**A**) Au nanorods (AuNRs), (**B**) AuNRs/PNIPAM electrospun fibers and (**C**) photograph of AuNRs/PNIPAM composite film immersed in water. Reprinted with permission from reference [97]. Copyright ACS Publications, 2017.

In this context, Zhang et al. [97] incorporated gold nanorods (AuNRs) into pNIPAM NFs in order to create a hybrid composite with fast thermal/optical response and structural integrity by electrospinning. They prepared a mixture of the pNIPAM polymer at 12 wt% and AuNRs in THF, then electrospinning was performed to achieve the hybrid AuNRs-IN-pNIPAM NFs. The photothermal property of these metal nanoparticles and the thermo-responsive property of pNIPAM were demonstrated. They obtained a NFs heating from room temperature to 34.5 °C after 1 s of laser application, and a further increase to 60 °C in 5 s of irradiation. Figure 8 shows TEM images of both the AuNRs dispersion and the hybrid NFs where the metal nanoparticles are incorporated onto the pNIPAM surface, a photograph of the hybrid composite is also included.

Poly (ε-caprolactone diol) based polyurethane solutions were used to synthesize NFs by electrospinning and they were combined with gold nanoparticles [110]. This nanoformulation was loaded with temolozolamide and was designed as a potential implant that allows a continued release of the antitumor drug for the treatment of glioblastoma multiforme. In fact, in U-87 MG human glioblastoma cell line this nanoformulation achieves a greater cell death overtime in contrast with free temozolamide (25% more) that practically does not modify the percentage of cell proliferation. In the same way as with electrospun NFs made of poly(allylamine hydrochloride) and polyamide 6 covered with MWCNTs, these NFs were also covered with gold nanoparticles for the detection of the pancreatic cancer biomarker CA19-9. In this case, lower biomarker detection thresholds were obtained with the use of gold nanoparticles (1.57 U mL^−1^) compared with the use of MWCNTs (1.84 U mL^−1^) [79].

## 3. Conclusions

Electrospinning is a technique used worldwide that allows the fabrication of polymeric NFs in the range of micro- and nanometers. Biocompatible and biodegradable synthetic polymers are essential structures that have improved chemotherapeutic treatments in the biomedical field. During the last years they have been extensively and specifically reported as systems with important benefits in important fields, including drug delivery and cancer treatments. In this review we have summarized recent advantage for the fabrication of NFs by electrospinning focused on drug delivery applications and cancer treatments (magnetic and plasmonic hyperthermia). Two methods are principally used for the fabrication of NFs by electrospinning: blend and coaxial electrospinning. Based on these methodologies, we reported recent approaches for the fabrication of drugs-IN-NFs with drug delivery and cancer treatment applications. We not only included a direct introduction of the drug dissolved within the polymer, nowadays, a very useful strategy is the incorporation of colloidal particles used as vehicles into the NFs during electrospinning. These particles are able to increase the amount of drug into the NFs to be released in a constant and controlled manner. In this sense, in this review we have also included polymers with stimuli-responsive behavior, obtaining NFs with the ability to increase the drug delivery capability in the function of an external stimulus (temperature or pH). Indeed, electrospinning also offers the possibility to fabricate hybrid NFs, structured as polymeric NFs with systems such as CNTs, graphene oxide, or even magnetic or metallic nanoparticles. Consequently, we also pointed out in the relevance of hybrid NFs in drug delivery improvements as well as in the reduction of solid tumors through magnetic and plasmonic hyperthermia.
